# Dynamics of DNA methylomes underlie oyster development

**DOI:** 10.1371/journal.pgen.1006807

**Published:** 2017-06-08

**Authors:** Guillaume Riviere, Yan He, Samuele Tecchio, Elizabeth Crowell, Michaël Gras, Pascal Sourdaine, Ximing Guo, Pascal Favrel

**Affiliations:** 1 Normandy University, Caen, France; 2 Université de Caen Normandie, UMR BOREA MNHN, UPMC, UCBN, CNRS-7208, IRD-207, Caen, France; 3 Ministry of Education Key Laboratory of Marine Genetics and Breeding, College of Marine Life Sciences, Ocean University of China, Qingdao, Shandong, China; 4 Haskin Shellfish Research Laboratory, Department of Marine and Coastal Sciences, Rutgers University, Port Norris, NJ, United States of America; University of Washington, UNITED STATES

## Abstract

DNA methylation is a critical epigenetic regulator of development in mammals and social insects, but its significance in development outside these groups is not understood. Here we investigated the genome-wide dynamics of DNA methylation in a mollusc model, the oyster *Crassostrea gigas*, from the egg to the completion of organogenesis. Large-scale methylation maps reveal that the oyster genome displays a succession of methylated and non methylated regions, which persist throughout development. Differentially methylated regions (DMRs) are strongly regulated during cleavage and metamorphosis. The distribution and levels of methylated DNA within genomic features (exons, introns, promoters, repeats and transposons) show different developmental lansdscapes marked by a strong increase in the methylation of exons against introns after metamorphosis. Kinetics of methylation in gene-bodies correlate to their transcription regulation and to distinct functional gene clusters, and DMRs at cleavage and metamorphosis bear the genes functionally related to these steps, respectively. This study shows that DNA methylome dynamics underlie development through transcription regulation in the oyster, a lophotrochozoan species. To our knowledge, this is the first demonstration of such epigenetic regulation outside vertebrates and ecdysozoan models, bringing new insights into the evolution and the epigenetic regulation of developmental processes.

## Author summary

Elucidating the mechanisms which govern the development of multicellular animals and their evolution is a fundamental task. Epigenetic mechanisms like DNA methylation have recently emerged as critical regulators of mammalian development through the control of genes that determine the identity of cells and the transmission of parental imprints. In invertebrates however, DNA is mostly unmethylated and does not play a role in development except in the peculiar case of social insects. Therefore the significance of DNA methylation in development is thought to be restricted to vertebrates, and thereby considered a recent evolutionary acquisition, and the situation in more distant organisms is unknown. Here we investigated the dynamics of genome-wide DNA methylation patterns in a mollusc, the oyster *C*. *gigas*, throughout its development. We found that the dynamics of DNA methylation correspond to the expression dynamics of distinct functional gene clusters that control two critical development steps, cleavage and metamorphosis, and we provide insights into the underlying molecular mechanisms in a non-vertebrate species. These findings challenge the present considerations on the evolution of developmental processes and their epigenetic regulation, and open a new area of research in molecular and developmental biology in invertebrates.

## Introduction

The methylation of DNA is a prevalent epigenetic mark that is deeply rooted in evolution and found from bacteria to mammals. Despite that metazoan organisms display methylation on cytosines, great variations exist in the amount and distribution of methylcytosines (meCs) across taxa. DNA methylation is an essential feature of mammalian development because meC patterns are associated with a wide range of cell processes whose subtle combination is required for the embryo to develop into a complex adult organism exhibiting differentiated cell types. In mammals, ca. 60 to 80% of CpG cytosines are methylated and exhibit mostly stable patterns across tissues. CpG rich regions (CpG islands) are prevalent at transcription start sites, and the methylation of promoters correlates to gene silencing during development [1]. CpG dinucleotides are overrepresented in promoters of development and housekeeping genes which are protected from methylation by transcription factor binding and subsequent DNA methyltransferase exclusion [2], reflecting poor methylation in the germline over evolutionary time. However, DNA methylation can be highly dynamic at precise locations during development, as illustrated by the demethylation wave observed in parental pronuclei, the epigenetic reprogramming of the germline or the differences between the epigenomes of germ and somatic cells [1, 3]. Consistently, DNA methylation shapes cell differentiation (reviewed in [4]) notably through silencing of pluripotency factors [5, 6] and of germline specific genes in somatic cells [7] at lineage commitment by *de novo* methylation. DNA methylation is also implicated in genome defence against transposable element activity [8], maintenance of parental imprints [9, 10], and X chromosome inactivation (review in [11]). Developmental processes are not only triggered by DNA methylation, whose causal role remains debated [12, 13], but by networks of epigenetic regulators including histone modifiers [14], non coding RNAs [15], transcription factors [16] and DNA methyltransferases [17, 18]. DNA methylation stabilizes the chromatin context underlying cell fate decisions that are propagated through cell generations by maintenance of the meC landscapes (review in [4]).

In invertebrates, DNA is much less methylated and meCs are not evenly distributed but exhibit mosaic patterns [19, 20]. DNA methylation in insect models is rare and mostly confined to gene bodies (gene body methylation, GBM) [20]. In hymenopterans, GBM controls exon selection[21] and governs important developmental outcomes such as caste differentiation in the honeybee [22, 23] and in ants [24, 25], as well as developmental gene expression in the wasp *N*. *vitripennis* [26, 27]. However, DNA methylation and its developmental significance seem essentially restricted to a peculiar evolutionary acquisition in hymenopterans. Indeed, in *Drosophila*, early genes are controlled by *cis*-regulatory elements, non-coding lnc- and miRNAs, and transcription factors including polycomb and trithorax complexes (review in [28]) but not by DNA methylation. The actual presence and function of meCs in the fruitfly genome have been under discussion, and the nematode *C*. *elegans* even lacks conserved DNA methylation machinery. Therefore DNA methylation is considered absent in the ecdysozoan common ancestor, in line with animal genomes evolving towards an overall loss of DNA methylation in protostomes [20] such as insects, compared to deuterostomes [29] such as mammals. As a consequence of this basic divergence between ‘methylated vertebrates’ and ‘unmethylated invertebrates’, and in spite of the tremendous variability of organisms and life traits within protostomes, DNA methylation is largely neglected outside insects.

However, recent studies in lophotrochozoans (that include molluscs and annelids), the sister group of ecdysozoans (that include insects and nematodes), suggest a more complex situation. Although meCs similarly exhibit a mosaic distribution, mollusc genomes are far more methylated than insects’, where methylated genomes display ca. 0.15% of meCs [30], whereas this value reaches ca. 2% in the snail *Biomphalaria glabrata* [31] and in the gills [32] and mantle [33] of the oyster *Crassostrea gigas*. In this bivalve of greatest ecological and economical importance, GBM is predominantand associated to mRNA content [32, 34]. Surprisingly, exposure to a DNMT inhibitor disrupts the oyster embryogenesis [35], and meCs are present in the promoter of some development genes with a direct influence on their expression [36]. These data point to developmental significance for DNA methylation in a lophotrochozoan species [37], challenging the current view on the evolution of epigenetic regulation of developmental processes.

Here, to shed light on this point, we provide the first characterisation, to our knowledge, of genome-wide DNA methylation dynamics covering the development of a lophotrochozoan species. Using a development stage-wise MeDIP-seq approach, we characterized the methylome dynamics from the egg to the completion of organogenesis in the oyster *C*. *gigas*. Epigenetic landscapes were analysed at both a global, physical and more local, feature-related scales, together with mRNA expression and functional annotation, and indicate a dynamic regulation of DNA methylation at critical developmental steps.

## Results

### DNA methylation landscapes reveal persistence of wide-scale maternal meC patterns as well as stage- and feature- dependent methylome dynamics marked at cleavage and metamorphosis

**Fig 1:**
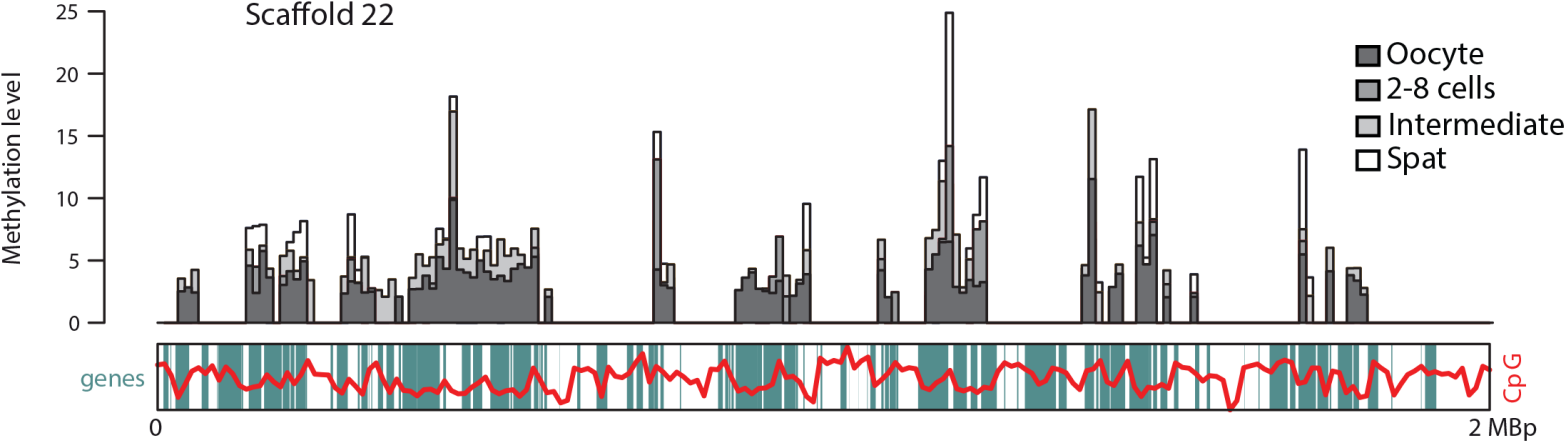
Large-scale methylation landscapes during oyster development reveal persistence of wide maternal methylation patterns and methylome dynamics. Physical methylation map of scaffold 22. The methylation level by 10 kbp windows (normalized counts) is plotted for oocyte, 2/8 cells, Intermediate (mean of morula, blastula, gastrula, trochophore and D larvae stages) and spat stages (dark grey to white) on the scaffold 22 gene map (gene positions are indicated as green boxes and GC content as a red line).

The methylated DNA immunoprecipitation followed by high throughput sequencing (MeDIP-seq) approach enabled genome-wide assessment of methylation and its variations during oyster development. Large-scale genome-wide methylome dynamics were investigated by analyses of differentially methylated regions (DMRs) and physical maps. DMRs highlighted 4 main developmental phases (oocytes, 2–8 cells, mid-larval, spat) separated by 3 main developmental steps: cleavage (C step), gastrulation and organogenesis (I step, intermediate) and metamorphosis (M step), respectively (S1A Fig).The morula, blastula, gastrula, trochophore and D-larva stages were grouped into an intermediate mid-larval stage, because DMRs and individual feature methylation profiles (see below) showed only minor differences. Physical methylation maps of genomic scaffolds confirm the mosaic characteristic of oyster DNA methylomes, which display a succession of methylated and non methylated regions bearing gene clusters of variable length with no obvious organisation or relationship to CpG content (Fig 1). Developmental methylation dynamics mostly affect regions that were already methylated in oocytes, with hypermethylation prevailing during development (Fig 1). Regions not methylated in oocytes mostly remain unmethylated and only little *de novo* methylation of previously unmethylated regions is observed that lie almost exclusively between two adjacent previously methylated regions or at their direct proximity (Fig 1). Indeed, 98.7% of the genes that are methylated at the spat stage were already methylated in oocytes. The mean distance from genomic features to the nearest DMR was drastically shorter at the M step (ca.5 kb for CDS to 20 kb for TE) than at other steps (ca. 100 to 200 kb at C and I), indicating that DNA methylation is more evenly regulated throughout the genome at the M step than in the C step (S1B Fig). Although DMR length did not exhibit marked variations, they were not equally distributed regarding genome features amongdevelopment steps. Many more DMRs were found at the C (n = 1043) and M (n = 2230) steps than at the I step (n = 14), with methylation being preferentially regulated in exons (CDS), repeats (REP) and transposable elements (TEs) (S1C Fig), and in class I TEs (i.e. retrotransposons) compared to class II TEs (i.e. DNA transposons) (Pearson’s *χ*^2^: p<0.0001***; C step: 122 DMRs in class I TEs vs. 81 in class II (60.1% vs. 39.9%); M step: 121 in class I TEs vs. 63 in class II (65.7% vs. 34.2%); genome, 64150 class I TEs vs. 21263 class II (75.1 vs.24.9%)).

**Fig 2:**
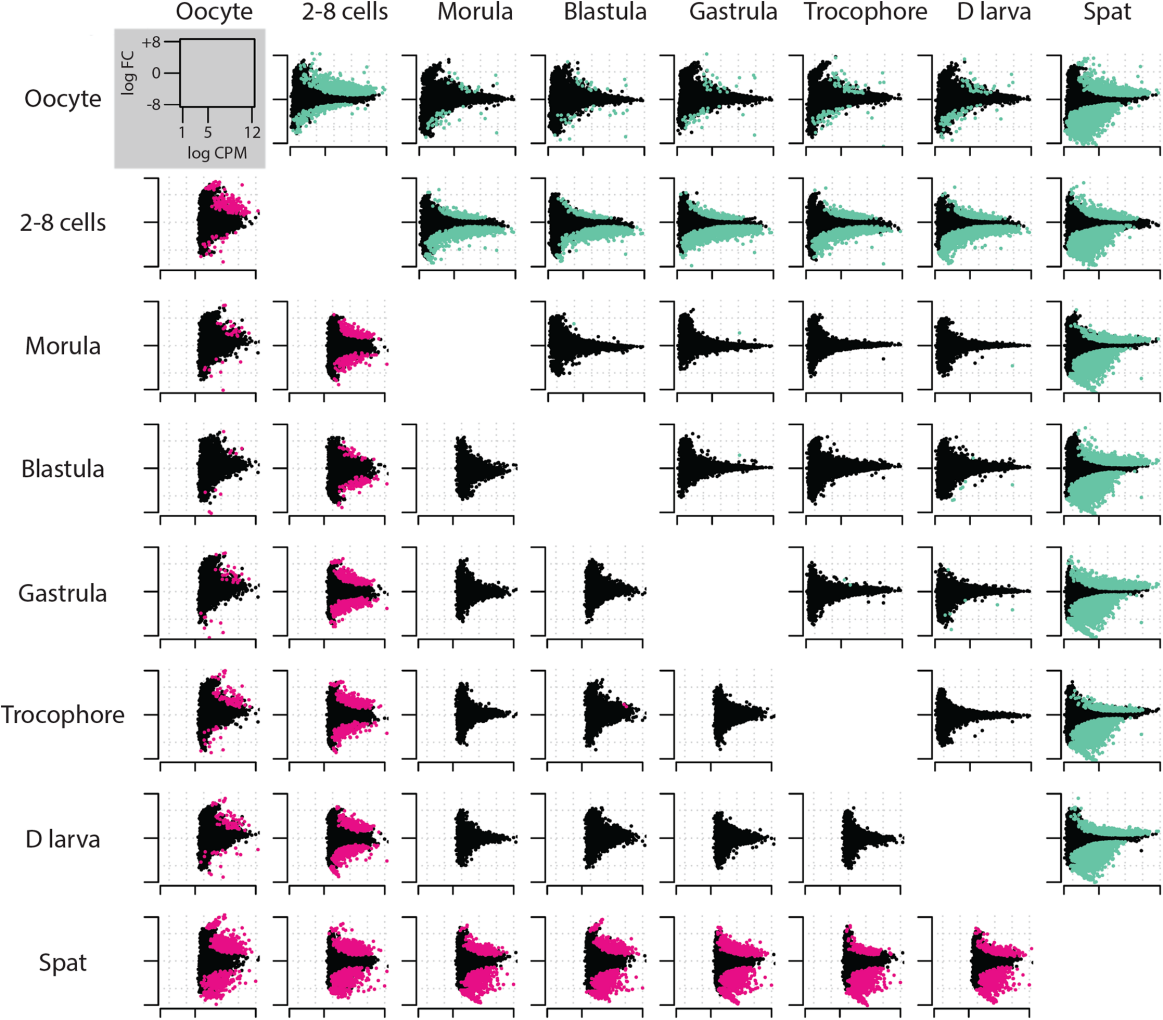
Oyster developmental methylomes exhibit feature- and stage-dependent landscapes highlighting cleavage and metamorphosis. Pairwise comparisons of the methylation of coding sequences (CDS, upper-right) and transposable elements (TE, lower-left) among development stages listed in the chronological order. Coloured points are significantly differentially methylated CDS (green) and TEs (pink) between stages (Student’s T test, p<0.01). The methylation level (log counts per million, x axis) and its variation (log fold change, y axis) are shown.

In parallel to genome-scale investigations, methylation landscapes were examined at the level of individual genomic features (i.e. exons (CDS), introns (INT), promoters (PRO), repeats (REP) and transposable elements (TE)). Most of the reads (81.5 ± 0.95%) mapped to the considered features and a great majority (ca. 90%) of methylation was found within gene bodies (CDS and INT, S4A Fig). Overall, the distribution of methylation depends on the development stage (Pearson’s *χ*^*2*^: p<2.10^−16^***, S2A Fig). The relative methylation of CDS is strongly increased after metamorphosis at the expense of the other features, especially introns (correlation between methylation in CDS and INT: Pearson R = -0.985, p<0.0001***), but not TEs (S2A Fig). This is because the intermediately methylated genes (ca. 2 to 6 log counts per million (CPM)) have their CDS markedly hypermethylated at the spat stage (Fig 2). The individual methylation level of an important number of TEs increases in 2–8 cell embryos,and the stability observed thereafter is due to compensation between individual TE hypermethylation and hypomethylation in spats (Fig 2). The biological coefficient of variation (BCV) analyses of feature methylation between development stages clearly discriminate the 2–8 cells and spat stages from one another and apart from the other stages, which are grouped and may display a gradual distribution regarding embryogenesis chronology (S4B Fig). The methylation profiles depend on the feature considered (S2C Fig) thereby confirming both the feature- and development stage-specificity of the dynamics of oyster DNA methylomes, especially marked at the 2–8 cells (cleavage) and spat (post metamorphosis) stages.

### Developmental kinetics of gene-body methylation are associated to transcription regulation

**Fig 3:**
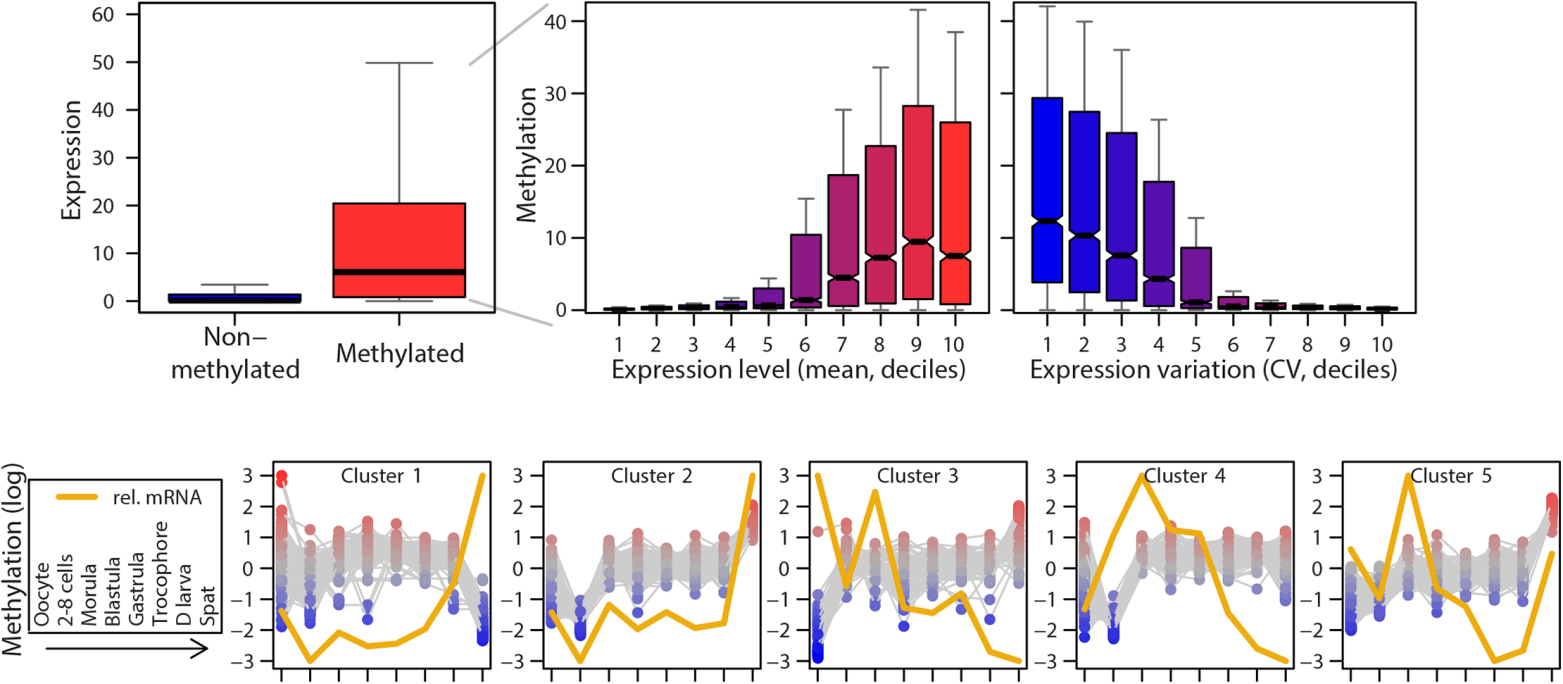
Methylation dynamics correspond to expression dynamics during oyster development. **a:**CDS methylation level is associated to expression level during development. Expression level of non-methylated and methylated genes (CDS methylation is shown, left). Relation between CDS methylation and mRNA levels (mean across development stages, deciles) of the methylated genes (middle). Relation between CDS methylation and expression variability of the methylated genes (coefficient of variation (CV) across development stages, deciles) (right). **b:** Developmental methylation (grey) and mRNA kinetics (yellow) of the 5 gene clusters obtained by K-means clustering of differentially methylated genes (1-way ANOVA of normalized CDS methylation against developmental stages, p<0.0001).

DMR proximity is associated to gene expression variability, and whether the DMR lies upstream or downstream has no influence (S3A Fig). Consistently, DMR-associated genes have their expression level more regulated than genes not associated to a DMR at each developmental step, although DMR and mRNA level variations were not correlated (S3B Fig). Compared to moderate changes, extreme methylation variations tend to hinder mRNA level regulation (S3B Fig). At a finer scale, most genes display a detectable methylation (20704 methylated genes vs. 7197 non methylated genes) during oyster development. The non methylated genes are mostly silent whereas the methylated genes are dramatically more expressed (Fig 3A). These genes have their mRNA level positively associated to their CDS methylation level, with a slight drop for genes within the 10^th^ expression decile. Conversely, the methylation level decreases with expression variability (Fig 3A). These results indicate that methylation marks highly and stably expressed genes. Although the exact localisation of methylcytosines is hampered by the resolution of MeDIPseq (ca. 250 bp), gene expression decreases with the hypermethylation of the INT or CDS feature over the other (ANOVA p<0.001), and the expression variability is correlated to the methylation pattern variability (p<2.10^−16^***) suggesting an optimal in-gene methylation pattern for maximum transcription (S4 Fig).A large set (26%) of oyster genes exhibits a dynamic CDS methylation during development (ANOVA across stages p<0.01**), and gene clusters can be discriminated based on their distinct CDS methylation kinetics (Fig 3B). However, methylation and mRNA level kinetics are correlated for only ca. 10% of these genes (r^2^>0, p<0.05*) (Fig 3B). All together, these results indicate that during oyster development, unmethylated DNA is associated to transcription repression whereas methylated DNA corresponds to gene expression, with the dynamics of gene-body methylation being associated with transcription regulation.

There was no association between PRO methylation and gene expression. The methylation level or coefficient of variation of gene features (PRO, CDS or INT) was not found to be correlated to the number of transcript variants during oyster development.

### DNA methylation dynamics affect functional gene clusters of the corresponding development steps

Gene ontology (GO) annotation of DMRs depends on the development step considered. Cleavage DMRs bear genes with functional annotation (Biological Process ontology) related to egg vitellogenic resource consumption, mRNA metabolism and nuclear genome processes, whereas metamorphosis DMR genes are enriched in terms related to transport within the cell and protein degradation (Table 1). The gene clusters based on methylation kinetics exhibit specific GO term distribution in ontologies (Biological Process, Molecular Function and Cell Component) and little GO terms in common (S5A Fig). Conversely, selected Biological Process ontology terms display specific methylation level and developmental dynamics (S5B Fig), indicating that methylation dynamics correspond to the distinct functional pathways related to specific steps of oyster development.

**Table 1:** Methylation dynamics and functional annotation.

	**GO.ID**	**Term**	**P-value**
**‘C’ DMRs**			
	GO:0006563	L-serine metabolic process	0.00076
	GO:0016192	vesicle-mediated transport	0.00081
	GO:0016071	mRNA metabolic process	0.00146
	GO:0000956	nuclear-transcribed mRNA catabolic process	0.00224
	GO:1901605	alpha-amino acid metabolic process	0.00289
	GO:0006520	cellular amino acid metabolic process	0.00421
	GO:0006401	RNA catabolic process	0.00441
	GO:0006402	mRNA catabolic process	0.00441
	GO:0006265	DNA topological change	0.00721
	GO:0034655	nucleobase-containing compound catabolic process	0.00743
**‘M’ DMRs**			
	GO:0044237	cellular metabolic process	0.00019
	GO:0006886	intracellular protein transport	0.00082
	GO:0034613	cellular protein localization	0.00114
	GO:0070727	cellular macromolecule localization	0.00114
	GO:0046907	intracellular transport	0.00208
	GO:0051649	establishment of localization in cell	0.00320
	GO:0009987	cellular process	0.00344
	GO:0051641	cellular localization	0.00413
	GO:0006511	ubiquitin-dependent protein catabolic process	0.00425
	GO:0019941	modification-dependent protein catabolic process	0.00425

Biological process annotation of ‘C’ (cleavage) and ‘M’ (metamorphosis) DMRs. The ten most enriched ontology terms and the corresponding p-value for enrichment (Fishers' method) are given.

## Discussion

The present work constitutes, to our knowledge, the first description of genome-wide methylome dynamics in a lophotrochozoan model. Their functional significance in oyster development brings new insights into the transcriptional regulation mediated by DNA methylation and the evolution of the epigenetic mechanisms underlying ontogenic processes in a lophotrochozoan species.

DNA methylation landscapes are highly dynamic during oyster ontogenesis and depend on the development stage (Fig 1), as suspected from variations of the global DNA methylation levels [35],. Furthermore, meC patterns also depend on genome features (CDS, INT, TE). DNA is mostly methylated within genes, and particularly in exons, in line with previous reports on oyster gills [32], mantle [33] or gametes [34]. Methylated coding sequences get hypermethylated during development at the expense of other genomic features, especially introns but not TEs. However, not all genes get hypermethylated (Fig 2), unravelling a precise and individual regulation of their DNA methylation (Fig 2 and S2C Fig). Such regulation is marked at cleavage and metamorphosis, assuming a participation of methylome dynamics at these two precise and critical development steps, as suggested by the altered phenotypes observed upon DNMT inhibition during oyster development [35].

The relationship between gene body methylation (GBM) and mRNA levels clearly indicates a biological significance of DNA methylation dynamics in gene expression during oyster development. Indeed, exon methylation is almost always required for transcription and marks stable and high expression (Fig 3A), in line with single time-point methylomes of adult tissues [32–34]. In addition, our study suggests that regardless of the level, the pattern of methylation within genes may be associated to transcriptional regulation. Indeed, a skewed GBM pattern corresponds to a diminished transcription, and the variability of methylation patterns and of mRNA levels are positively correlated (S4 Fig), in spite of the fact that the limited resolution of MeDIP-seq does not allow a more precise localisation of meCs within exons and introns. Such an influence is reported here for the first time to our knowledge and supports previous hypotheses of GBM increasing ‘transcriptional opportunities’ of methylated genes [38]. However the methylation of gene features was not associated to the number of transcript variants. This unexpected finding does not substantiate a role for methylation in exon selection in the oyster, in contrast to insects [21] and to previous hypotheses [33], although higher resolution methylomes might be needed to clarify this point.

In addition to transcriptional regulation, this work shows that methylation dynamics are associated with developmental stage-specific functional pathways in the oyster. Indeed, GBM dynamics define gene clusters with specific functional annotation (Fig 3 and S5 Fig). Consistently, DMR annotations at cleavage are clearly relevant to egg mRNA and vitellogenic resource consumption as well as nuclear genome processes (Table 1), bringing epigenetic indications of early zygotic genome activation in the oyster. The requirement of transcription for the cleavage in the distant annelid, the leech *Helobdella* [39] suggests that this situation could be general in lophotrochozoans. It implies stabilisation of open chromatin states at loci of cleavage genes that lie within DMRs during oyster development. Conversely, persistent unmethylated regions could be of functional significance for developmental gene silencing [40] as reflected by the strong repression of unmethylated genes (Fig 3A). The primary resemblance with oocyte DNA methylation patterns (Fig 1) suggests that these loci could be inherited, their transcription being further regulated by local methylation dynamics in the embryo, reminiscent of recent findings in the mouse [41]. Oysters exhibit a mosaic development and the fate of their blastomeres is determined during early ontogenesis notably through specific transcriptomes [42]. Besides, the oyster genes with maximum sequence similarity to vertebrate pluripotency factors Pou5f1 and Sox2 (GenBank accession number CGI_10005968 and CGI_10010085, respectively) display their highest embryolarval methylation at cleavage (S1 File). This suggests that DNA methylation could link zygotic genome activation and cell differentiation [43], consistent with the hypothesis that methylome dynamics participate in both cell differentiation and epigenetic memory in the oyster. Surprisingly, gastrulas and later mid-larval stages display little methylation difference despite dramatic morpho-physiological changes such as organ and shell formation, larval growth and the onset of digestion. Such processes are likely governed by other factors, such as *Hox* genes and Polycomb repressive complex orthologues [36], although target genes should lie in chromatin competent for transcription. Long intergenic non-coding RNAs (lincRNAs) were recently hypothesised to play a role during oyster development [44], but they are mostly expressed late after metamorphosis and therefore unlikely contribute to mid-larval state epigenetic control. The ‘M’ step DMRs are more abundant and widespread across the genome (Fig 1 and S1 Fig), further supporting the association of distinct methylation landscapes with the transcriptomes of the various cell types within post-metamorphosis oyster larvae. Their functional annotation related to transport and targeted protein degradation evokes the importance of cell morphology changes related to metamorphosis.

Transposable elements can be either hypermethylated or hypomethylated after metamorphosis (Fig 2) independently of being retro- or DNA transposons. Some young repetitive elements such as SINEs are preferentially methylated [33] and may still be active in the oyster genome [42]. Therefore DNA methylation may be important for TE control and genome plasticity during oyster development, unlike most ecdyzosoans [23, 27] but like vertebrates [45]. Whether DNA methylation facilitates SINE transcription is unknown, but retrotransposons are more associated to DMRs, raising the possibility that methylation influences their transcription, which may be silenced by other regulators such as piRNAs [46].

The precise molecular pathways associating transcription regulation with DNA methylation dynamics in the oyster remain unknown. Nevertheless, taken altogether our results raise the assumption that DNA methylation locally impairs chromatin compaction, and propagates transcription-competent states outwards to flanking unmethylated sequences. In this context, initiation may be hampered by promoter methylation [36], and elongation by too high and/or inconsistent meC density within gene bodies. This hypothesis does not contradict the situation in vertebrates where high meC levels inhibit transcription initiation [47]. Indeed, vertebrate genomes are highly methylated whereas methylation of oyster DNA is scarce. Then, both high meC levels in the oyster and meC depletion in vertebrates would correspond to moderate methylation levels allowing transcription. It remains to be determined whether DNA methylation is a cause or a stabiliser of transcriptional regulation, or both, which is under debate [12, 13]. The association between GBM and expression could also be explained by increased accessibility of transcribed DNA to methyltransferases [47, 48].

This study brings new insights into the evolution of the epigenetic regulation of developmental processes. Regarding the DNA methylation in development, oysters resemble deuterostomes more than other protostomes. Therefore such epigenetic regulation could be present in the bilaterian common ancestor and might be an ancient trait of metazoan organisms that would have been generally lost in ecdysozoans. Besides, there is poor consistency over evolutionary time in the methylation level of both oyster development genes and environment-response genes [49]. Such resemblance may suggest that development is an inducible biological process rather than a fixed program. Stochastics of ontogeny may thus be under environmental inputs implicating epigenetic mediation, and their reproducibility could result from probability canalisation by biological systems over evolutionary time. Future work towards the understanding of the interactions between epigenetic marks and chromatin dynamics across evolutionary lineages, as well as insights into the causal and/or stabilising role of DNA methylation in this context and upon environmental inputs, is required to decipher this issue.

### Conclusion

The present work constitutes, to our knowledge, the first description of genome-wide methylome dynamics in a lophotrochozoan model. Dynamics of DNA methylation in gene bodies are associated with transcriptional regulation, and the control of transposable elements may imply DNA methylation. The shifts in methyl DNA profiles and their functional outcomes are prevalent at cleavage and metamorphosis, and suggest the importance of inherited methylomes. These results demonstrate that DNA methylation dynamics underlie *Crassostrea gigas* development. The developmental significance of gene body methylation in the oyster brings new insights into the epigenetic regulation of developmental processes and its evolution.

## Materials and methods

### Animals

Oyster embryos were obtained as previously described [50]. Wild individuals were collected in Marennes- Oléron, France in August 2008 then transferred in mesh bags in February 2009 to Paimpol (northern Brittany, France, 48°48’ 24.49”N, 3° 0’ 22.84”W) until February 2010 and then to the Ifremer grow-out farm located at Aber-Benoît (northern Brittany, France, 48° 34’ 29.976”N, 4°36’ 18.378”W). These animals were exposed to disease during the spring of 2009 and suffered ca. 75% mortality. In April 2010 (Experiment 1 and 2) and February 2011 (Experiment 3), 60 individuals were transferred to the Ifremer marine station located at Argenton (Brittany, France, 48° 31’ 16.320”N, 4°46’ 01.998”W) for broodstock conditioning (6 weeks in 500 L flow-through tanks with UV-treated and 1 μm filtered seawater (TSW) at 19°C, enriched with a 1:1 in dry weight mixture of *Isochrysis affinis galbana* and *Chaetoceros gracilis* corresponding to a daily diet of a ration equivalent to 6% of the oyster dry weight). Diploidy of oysters was confirmed by flow cytometry of gill cells from randomly sampled animals as previously described [51]. Gametes from mature specimen (13♂ and 27♀, 10♂ and 24♀, 13♂ and 21♀, Experiment 1, 2 and 3 respectively) were obtained by stripping and filtered on a 100 μm mesh for the removal of large debris. For females, oocytes were harvested as the remaining fraction on a 30 μm mesh; for males, spermatozoa were harvested as the passing fraction through a 30 μm mesh. Oocytes were pre-incubated in TSW then mixed in a 5 L jar at 50–100 spermatozoids per oocyte (22 November 2010, 5 January 2011 and 12 April 2011 for experiment 1, 2 and 3 respectively). The embryonic development was completed in TSW in oxygenated 150 L tanks at 21°C for 48 h. The D-larvae were then collected and reared in flow- through rearing systems at 25°C. At the end of the pelagic phase (16 d), competent larvae were collected on a 225 μm sieve and allowed to settle on cultch. Post-larvae were maintained in downwelling systems where they were continuously supplied with enriched seawater. After 10 d, the spat were collected on 400 μm mesh. In the larval and post-larval stages, the oysters were fed the same diet as the broodstock. Throughout this time, the oysters were free of any abnormal mortality and OsHV-1 virus. Embryos were left unattended until sampling, i.e. before fertilization for control oocytes, and ca. 1 hour post-fertilization (hpf) for 2–8 cells stage, ca. 3 hpf for morulae, ca. 6 hpf for blastulae, ca. 9 hpf for gastrulae, ca.16 hpf for trochophore larvae, and ca. 24 hpf for D larvae. Spat was collected at 26 days, after settlement and metamorphosis. Developmental stages were assayed by microscopic observation based on morphological and motility criteria before and after fixation using 70% ethanol. Samples were split in aliquots of 2 million larvae, stored dry at -80°C and thawed only once before use. Each development stage was sampled from three distinct fertilization experiments (experiments 1, 2 and 3).

### DNA extraction and fractionation

Genomic DNA from ca. 2 million larvae per sample was purified by affinity chromatography (Macherey Nagel) following the manufacturer’s instructions. Degradation of contaminating RNA was realized using RNAse. DNA purity and concentration were assayed by spectrometry (Nanodrop, Thermo) and on-chip gel electrophoresis (Tape Station 2200, Agilent). DNA was sheared in ca. 250 bp fragments using a Covaris S2 sonicator (duty cycle: 10%, intensity: 4, cycles: 200, time: 80s).

### Library construction, Methyl-DNA immunoprecipitation (MeDIP) and sequencing

Twenty samples (n = 2 to 3 biological replicates per development stages) were processed for MeDIP-seq library preparation following the protocol of Taiwo et al. [52]. Briefly, 5μg DNA from each sample were used for DNA end-repair and dA-tailing (NEBNext reagents, New England Biolabs). Immunoprecipitation of methylated DNA (MeDIP) was realised on 1 μg DNA after end-repair, dA-tailing and purification using the MagMeDIP kit (Diagenode) using the manufacturer’s instructions. All DNA purifications were carried out using Ampure XP magnetic beads (Beckman Coulter) according to the recommended procedure. Ten randomly chosen samples were assayed following the manufacturer’s recommendations for MeDIP specificity with a mean value of 96.9%. Immunoprecipitated DNA was then amplified using Phusion DNA polymerase (New England Biolabs) and purified. After size selection and quality control, libraries were submitted to 2x76 bp paired-end sequencing using a GAIIx sequence analyzer (Illumina). This strategy produced ca. 80 million paired end reads i.e. 3.6±0.25x10^6^ reads per sample among which 80.7±0.1% were aligned to the genome, giving a 24.8±3.3-fold mean coverage.

### Data analyses

Data were analysed using a using a combination of dedicated R (bioconductor.org) and bash script as well as in-house R, bash, TiCL and PERL scripts. Data source files (NCBI project PRJNA324546) and scripts used for analyses are publicly available (github.com/BOREA-UNICAEN/MeDIPSeq-Dev-Gigas). Primary analysis was performed with RTA (Illumina) with default parameters and reads were demultiplexed using CASAVA v.1.8. Bases with a QC>30 were retained for further analyses. Paired-end reads were mapped to the oyster genome (assembly v.9) using BWA with default parameters and pair-sorted. Paired reads mapping to the following genomic features: exons (CDS), introns (INT), promoters (PRO), repeats (REP) and transposable elements (TE) [34] were counted using *HTseq-count* [53]. Only promoter sequences longer than 100 bp were retained for further analyses and ambiguous read pairs were discarded.

#### Differentially methylated regions (DMR) analyses

The analysis of differentially methylated regions was performed using *diffREPs* [54] (negative binomial test). Because of limitations due to the high number of scaffolds and the short length of many of them in the present genome assembly, the analyses were performed on the 2000 longest scaffolds (length > 44 kb), covering 93.4% of the total genome sequence. Comparisons were performed between each stage and the previous one in the chronological order of development. Morula, blastula, gastrula, trochophore and D-larva stages were grouped into an ‘intermediate larval phase’ according to results from pairwise-comparisons and preliminary DMR analyses showing only weak differences among their methylomes. DMRs were defined as regions of at least 1000 bp displaying at least a 2-fold difference between considered groups with an exact T-test (cut-off: p<0.0001), and only regions with a p-value<10^−7^ were retained for further analyses. Identified DMRs not mapping to a feature were not annotated. DMRs were annotated as corresponding to a feature only if completely covering the considered feature (i.e. feature start and stop positions included within the DMR). Distances between DMRs and features on the same scaffolds are given relative to their central position because no difference was observed when computing distances between features and DMR ends. The influence of DMR proximity on gene expression was computed with respect to the gene orientation (+/- strand). Functional analyses of DMRs were realised using *TopGO* [55] based on the annotated genes within DMRs.

#### Methylation level and dynamics of genomic features and individual feature elements

The methylation level was estimated as the number of reads corresponding to a designed feature element, per million reads (cpm) using *EdgeR* [53]. Only feature elements presenting at least one cpm in at least two biological replicates of the same development stage were considered methylated. In-gene methylation pattern was estimated using the INT/CDS methylation counts ratio, after normalization regarding the length of the considered elements. The distribution of methylation in features between development stages was tested using Pearson’s chi square test. Variations in the methylation level of individual feature elements were tested using two-tailed exact Student’s T test (pairwise comparison between stages, p<0.01 was considered significant) and ANOVA against developmental stages followed by Bonferroni’s post hoc test (variations across development stages, p<0.01 was considered significant). MDS-BCV analyses were realised using *EdgeR* [53]. Methylome landscapes were plotted only for significantly differentially methylated feature elements during development, using log transformed and normalised values of the methylation level. TEs and genes with significantly differentially methylated exons or introns according to this selection (n = 2133) were clustered regarding their methylation level using K-means clustering (S2C Fig).

#### Relationship between methylation, expression, transcript variants and functional annotation

Gene expression levels are from Zhang et al. [42] (S2 Table and S1 File). The GigaTON database [56] was used for additional analyses such as filtering of maternal or development-stage specific mRNAs and transcript variant number computing. The consistency of the methylation pattern in genes was computed as the ratio of INT and CDS methylation after cpm normalisation regarding feature lengths. Functional profiles were inferred from Gene Ontology annotations and analysed using *TopGO* [55]for specific profiles and term enrichment using Fisher’s method.

## Supporting information

S1 Fig DMR analyses during oyster development reveal wide-scale methylome dynamics. **a:** Schematic representation of DMR analyses across three distinct development steps: the ‘C’ step (cleavage): transition between the oocyte and 2/8 cells stages; the ‘I’ step (intermediate) between the 2/8 cells stage and the collection of morula, blastula, gastrula, trochophore and D larvae stages; and the ‘M’ step (metamorphosis) between the larval life and spat stages. **b**: Feature distance to nearest DMR in C, I and M steps. **c**: DMR annotation in C, I and M steps (CDS, green; INT, blue; PRO, light green; REP; purple; TE, pink; not annotated, white) with the genome as comparison.
(TIF)

S2 Fig Oyster developmental methylomes are feature- and stage-dependent and highlight cleavage and metamorphosis. **a:** Distribution of methylation within genomic features (CDS, exons; INT, introns; PRO, promoters; REP, repeats; TE, transposable elements) given as the proportion of reads mapped at each development stage. **b:** MDS/BCV plots of the methylation of transposable elements (TE, pink) and exons (CDS, green) at different developmental stages. **c:** Methylation landscapes of genes significantly differentially methylated in exons (CDS), in introns (INT), and of transposable elements (TE) across development from oocytes (dark grey) to spat (light grey) (1-way ANOVA of normalized methylation counts against developmental stages, p<0.0001). The normalised methylation level (low, blue; high, red) is shown in 3D heat maps.
(TIF)

S3 Fig DMR dynamics correspond to expression dynamics during oyster development. **a:** DMR proximity and gene expression variation in C (left), I (middle) and M (right). The colour represents the number of genes (low, blue; high, red) and the distance considered is from the nearest DMR with respect to genes orientation. **b:** DMR methylation variation and gene expression variation in C (left), I (middle) and M (right). Colours indicate the expression change (upregulation, red; downregulation, blue; no change, grey). The box represents the expression level variation of genes not associated with DMRs for comparison.
(TIF)

S4 Fig GBM pattern is associated to mRNA expression. Relationship between in-gene methylation pattern (INT/CDS methylation ratio) and mRNA level (left). Relationship between methylation pattern variation (INT/CDS methylation ratio CV) and mRNA level CV (right).
(TIF)

S5 Fig Methylation dynamics and functional annotation. **a:** Gene clusters based on developmental methylation kinetics have specific functional annotation. Gene ontology annotation of each cluster in Fig 3B is represented by boxes (sectors) (BP, biological process, heavy blue; MF, molecular function; light blue; CC, cell component, green). The box height is proportional to the number of terms. The width of the links indicates the proportion of common terms between gene cluster annotations. **b**: Selected ontology terms display specific methylation dynamics during oyster development. The p-value for enrichment test is given for each indicated term at each development stage (0.1<p<1, light grey; 0.05<p<0.1, blue; p<0.05, dark grey) (left). The methylation profile (blue, low; red, high), the number of genes annotated with the indicated ontology term (middle), and their mean methylation level across development (right) are indicated.
(TIF)

S1 Table Gene ontology annotation of gene clusters defined by methylation dynamics. The five most enriched terms are indicated for each ontology category (biological process, BP, molecular function, MF and cell component, CC).
(DOCX)

S2 Table Correspondence between development stages for analyses of DNA methylation (this study) and mRNA expression (Zhang et al, 2012, [42]). The RNAseq counts in Zhang et al. (RPKM values given in Table S14 of that paper) [42] were averaged as indicated.
(DOCX)

S1 File Expression values of genes during oyster development. The table contains the mRNA levels as computed using the RPKM values from the oyster genome project (Zhang et al. 2012 [42]) according to the correspondence given in S2 Table. The log fold change of mRNA expression between developmental steps (Cleavage, Intermediate and Metamorphosis, see text) is given.
(XLSX)
