# Supplementary figures and images for: Dynamics of DNA methylomes underlie oyster development
Source: PLoS Genet. 2017 Jun 8;13(6):e1006807. doi: 10.1371/journal.pgen.1006807 (PMC5481141; doi:10.1371/journal.pgen.1006807)

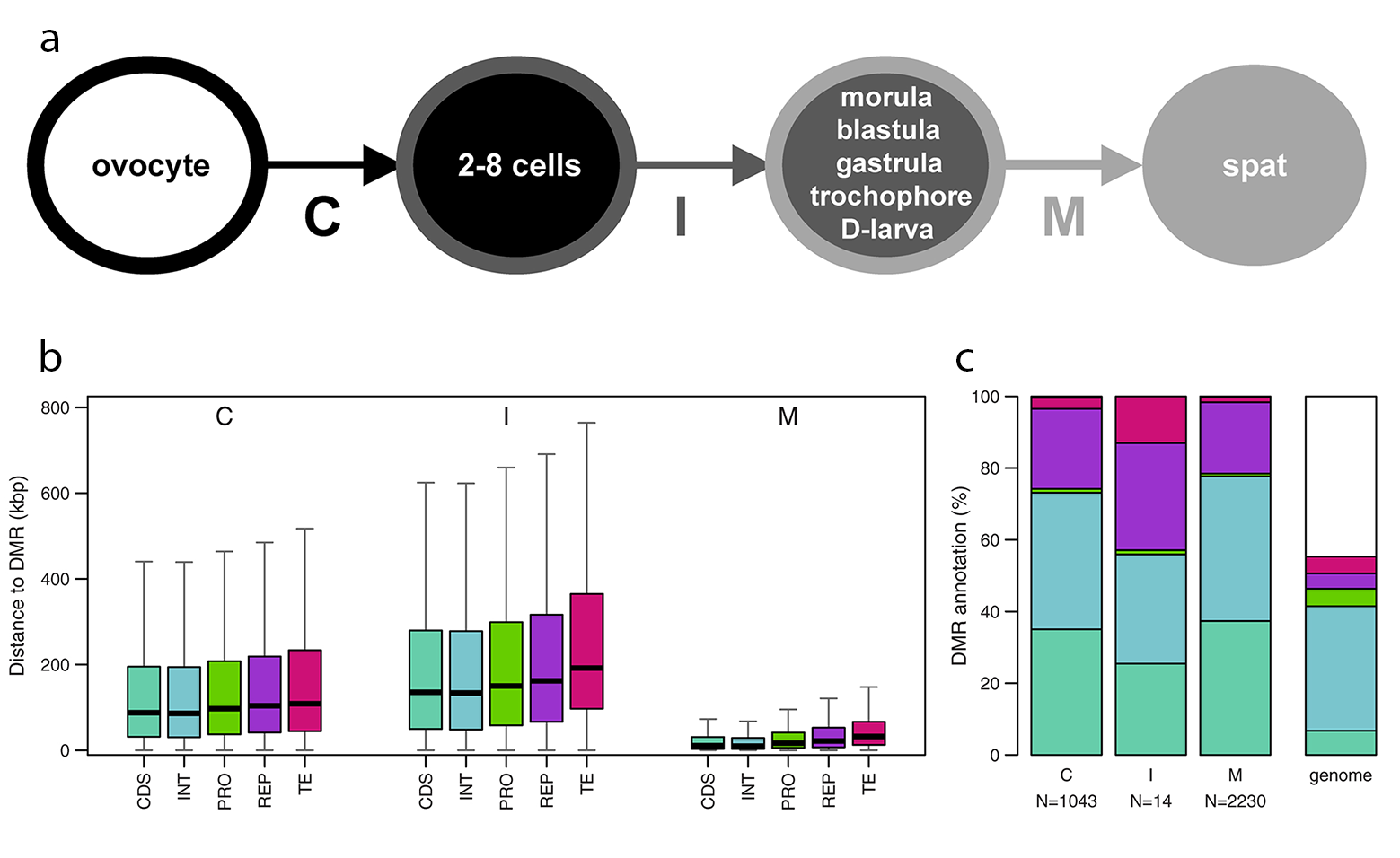

Supplement: S1 Fig — a: Schematic representation of DMR analyses across three distinct development steps: the ‘C’ step (cleavage): transition between the oocyte and 2/8 cells stages; the ‘I’ step (intermediate) between the 2/8 cells stage and the collection of morula, blastula, gastrula, trochophore and D larvae stages; and the ‘M’ step (metamorphosis) between the larval life and spat stages. b: Feature distance to nearest DMR in C, I and M steps. c: DMR annotation in C, I and M steps (CDS, green; INT, blue; PRO, light green; REP; purple; TE, pink; not annotated, white) with the genome as comparison. (TIF) [file pgen.1006807.s001.tif]

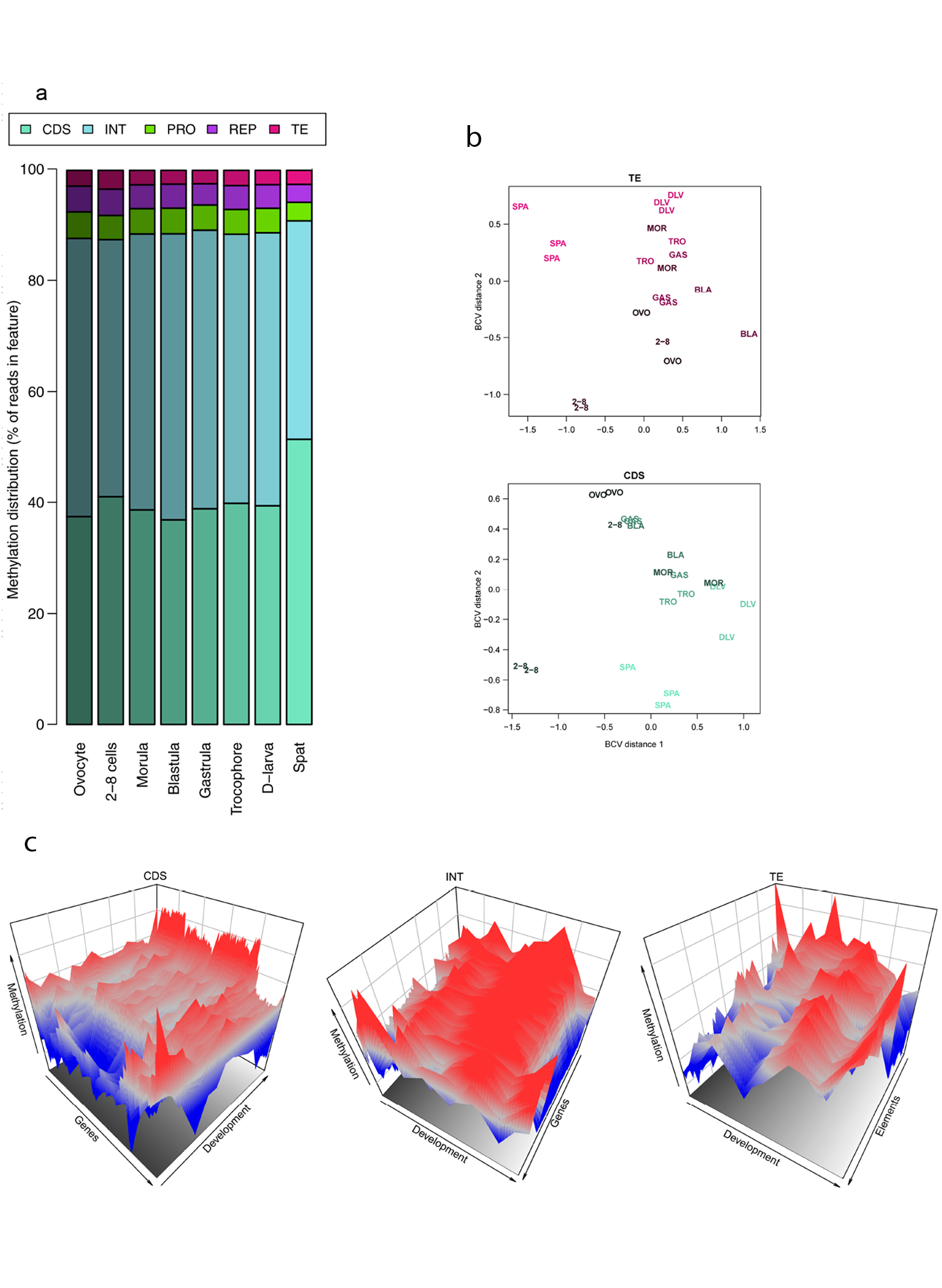

Supplement: S2 Fig — a: Distribution of methylation within genomic features (CDS, exons; INT, introns; PRO, promoters; REP, repeats; TE, transposable elements) given as the proportion of reads mapped at each development stage. b: MDS/BCV plots of the methylation of transposable elements (TE, pink) and exons (CDS, green) at different developmental stages. c: Methylation landscapes of genes significantly differentially methylated in exons (CDS), in introns (INT), and of transposable elements (TE) across development from oocytes (dark grey) to spat (light grey) (1-way ANOVA of normalized methylation counts against developmental stages, p<0.0001). The normalised methylation level (low, blue; high, red) is shown in 3D heat maps. (TIF) [file pgen.1006807.s002.tif]

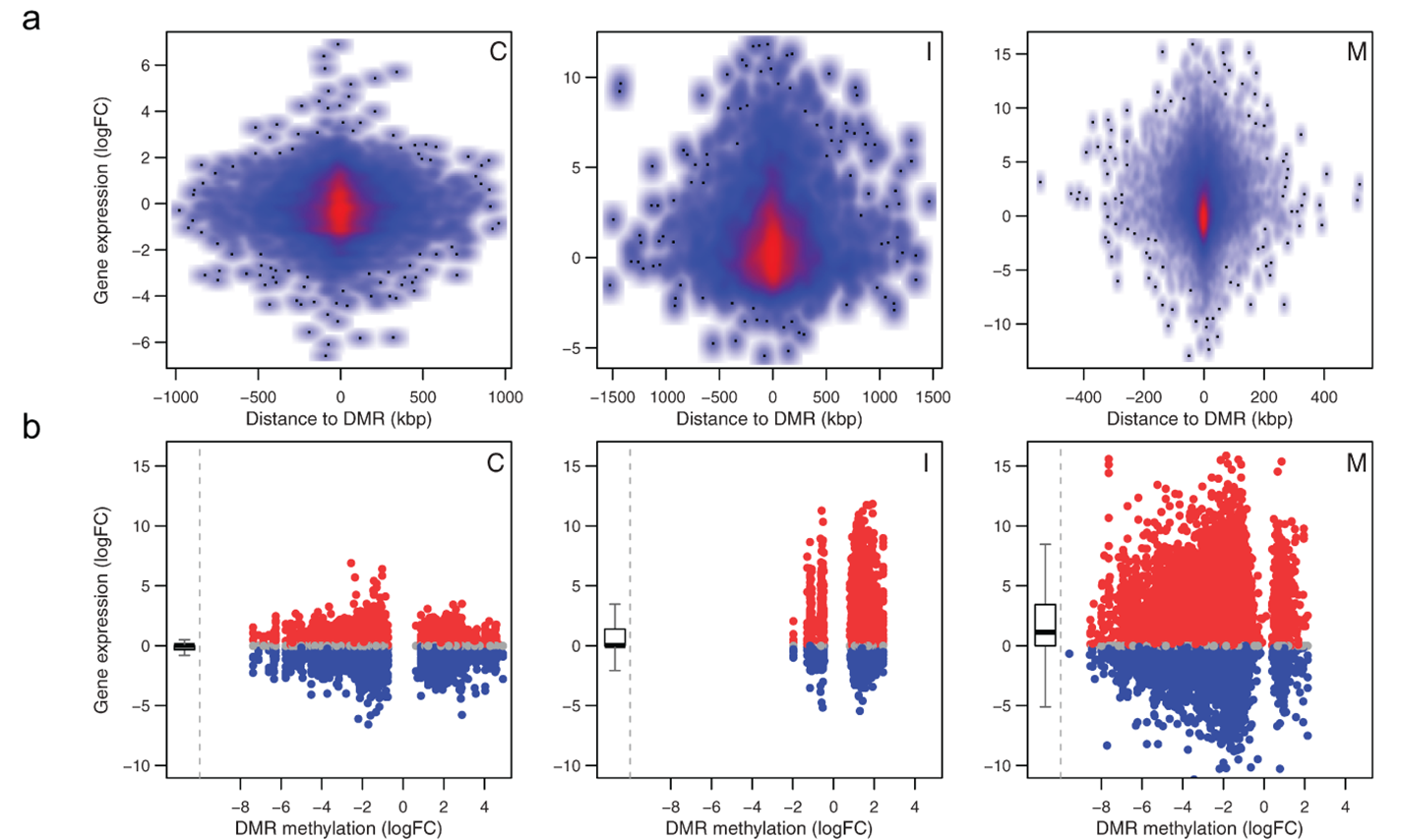

Supplement: S3 Fig — a: DMR proximity and gene expression variation in C (left), I (middle) and M (right). The colour represents the number of genes (low, blue; high, red) and the distance considered is from the nearest DMR with respect to genes orientation. b: DMR methylation variation and gene expression variation in C (left), I (middle) and M (right). Colours indicate the expression change (upregulation, red; downregulation, blue; no change, grey). The box represents the expression level variation of genes not associated with DMRs for comparison. (TIF) [file pgen.1006807.s003.tif]

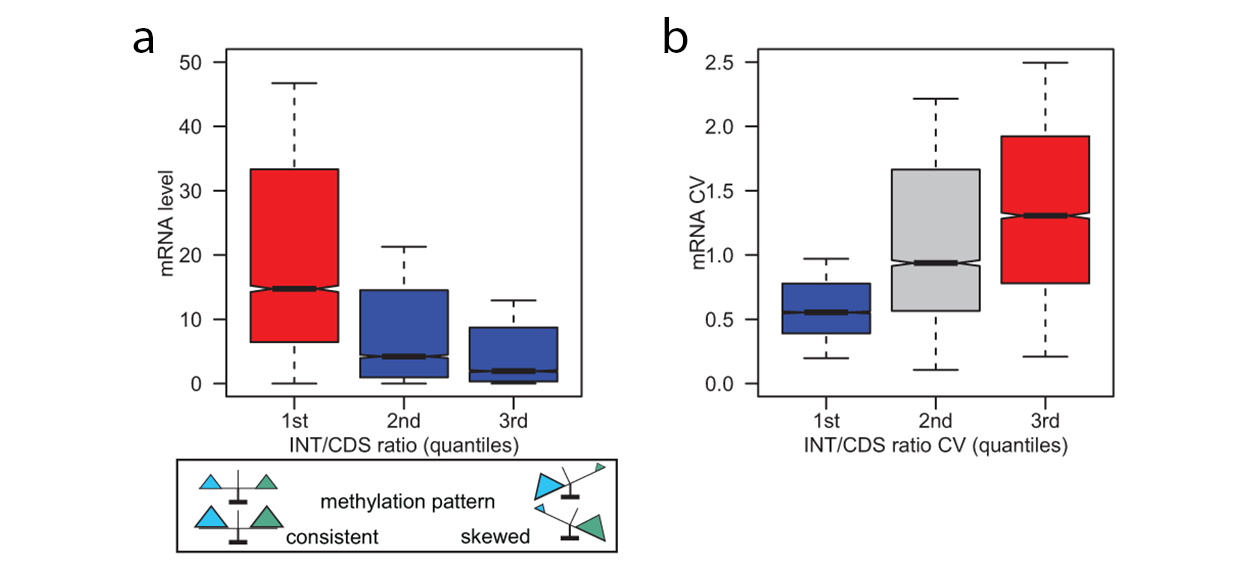

Supplement: S4 Fig — Relationship between in-gene methylation pattern (INT/CDS methylation ratio) and mRNA level (left). Relationship between methylation pattern variation (INT/CDS methylation ratio CV) and mRNA level CV (right). (TIF) [file pgen.1006807.s004.tif]

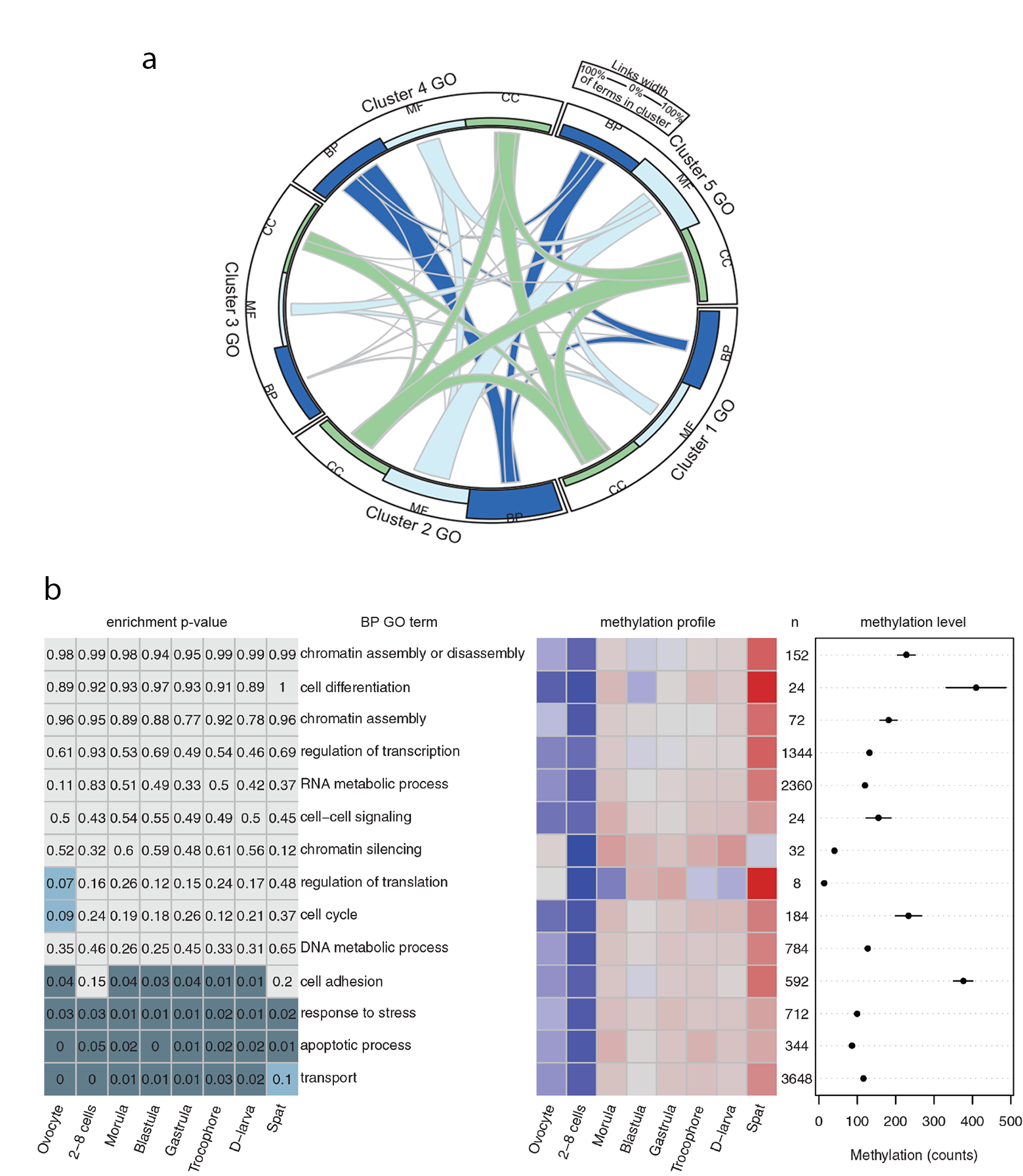

Supplement: S5 Fig — a: Gene clusters based on developmental methylation kinetics have specific functional annotation. Gene ontology annotation of each cluster in Fig 3B is represented by boxes (sectors) (BP, biological process, heavy blue; MF, molecular function; light blue; CC, cell component, green). The box height is proportional to the number of terms. The width of the links indicates the proportion of common terms between gene cluster annotations. b: Selected ontology terms display specific methylation dynamics during oyster development. The p-value for enrichment test is given for each indicated term at each development stage (0.1<p<1, light grey; 0.05<p<0.1, blue; p<0.05, dark grey) (left). The methylation profile (blue, low; red, high), the number of genes annotated with the indicated ontology term (middle), and their mean methylation level across development (right) are indicated. (TIF) [file pgen.1006807.s005.tif]
