# Supplementary material for: Dynamics of DNA methylomes underlie oyster development
Source: PLoS Genet. 2017 Jun 8;13(6):e1006807. doi: 10.1371/journal.pgen.1006807 (PMC5481141; doi:10.1371/journal.pgen.1006807)
Supplement: S1 Table — The five most enriched terms are indicated for each ontology category (biological process, BP, molecular function, MF and cell component, CC). (DOCX) [file pgen.1006807.s006.docx]

| Cluster | BP GO ID | BP annotation | MF GO ID | MF annotation | CC GO ID | CC annotation |
| --- | --- | --- | --- | --- | --- | --- |
| 1 | GO:0044237 | Cellular metabolic process | GO:0004518 | Nuclease activity | GO:0005622 | Intracellular |
| 1 | GO:0043170 | Macromolecule metabolic process | GO:0003824 | Catalytic activity | GO:0005623 | Cell |
| 1 | GO:0044260 | Cellular macromolecule metabolic process | GO:0004527 | Exonuclease activity | GO:0044454 | Cell part |
| 1 | GO:0044238 | Primary metabolic process | GO:0016788 | Hydrolase activity, acting on ester bonds | GO:0044424 | Intracellular part |
| 1 | GO:0008152 | Metabolic process | GO:0008833 | Deoxyribonuclease IV (phageT4-induced) activity | GO:0044432 | Endoplasmic reticulum part |
| 2 | GO:0006886 | Intracellular protein transport | GO:0005524 | ATP-binding | GO:0044424 | Intracellular part |
| 2 | GO:0046907 | Intracellular transport | GO:0032559 | Adenyl-ribonucleotide binding | GO:0005622 | Intracellular |
| 2 | GO:0034613 | Cellular protein localization | GO:0030554 | Adenyl nucleotide binding | GO:0032991 | Macromolecular complex |
| 2 | GO:0070627 | Cellular macromolecule localization | GO:0005515 | Protein binding | GO:0043234 | Protein complex |
| 2 | GO:0044237 | Cellular metabolic process | GO:1901363 | Heterocyclic compound binding | GO:0005623 | Cell |
| 3 | GO:0006066 | Alcohol metabolic process | GO:0008108 | UDP-glucose: hexose-1-phosphate uridylyltransferase activity | GO:0005643 | Nuclear pore |
| 3 | GO:1901615 | Organic hydroxyl compound metabolic process | GO:0070569 | Uridylyltransferase activity | GO:0046930 | Pore complex |
| 3 | GO:1901575 | Organic substance catabolic process | GO:0017056 | Structural constituent of nuclear pore | GO:0005635 | Nuclear envelope |
| 3 | GO:0009056 | Catabolic process | GO:0008889 | Glycerophosphodiester phosphodiesterase activity | GO:0043234 | Protein complex |
| 3 | GO:0044281 | Small molecule metabolic process | GO:0030234 | Enzyme regulator activity | GO:0031967 | Organelle envelope |
| 4 | GO:0044723 | Single-organism carbohydrate metabolic process | GO:0004190 | Aspartic-type endopeptidase activity | GO:0012505 | Endomembrane system |
| 4 | GO:0030258 | Lipid modification | GO:0070001 | Aspartic-type peptidase activity | GO:0044444 | Cytoplasmic part |
| 4 | GO:0048193 | Golgi vesicle transport | GO:0016836 | Hydro-lyase activity | GO:0098588 | Bounding membrane of organelle |
| 4 | GO:0006906 | Vesicle fusion | GO:0005515 | Protein binding | GO:0031090 | Organelle membrane |
| 4 | GO:0016050 | Vesicle organization | GO:0004363 | Glutathione-synthase activity | GO:0005737 | Cytoplasm |
| 5 | GO:0016310 | Phosphorylation | GO:0030554 | Adenyl-nucleotide binding | GO:0030286 | Dynein complex |
| 5 | GO:0006468 | Protein phosphrylation | GO:0005524 | ATP binding | GO:0044430 | Cytoskeletal part |
| 5 | GO:0044267 | Cellular protein metabolic process | GO:0032559 | Adenyl ribonucleotide binding | GO:0044422 | Organelle part |
| 5 | GO:0006796 | Phosphate-containing compound metabolic process | GO:0016887 | ATPase activity | GO:0044446 | Intracellular organelle part |
| 5 | GO:0006793 | Phosphoric metabolic process | GO:0017076 | Purine nucleotide binding | GO:0005875 | Microtubule-associated complex |
