# Supplementary material for: Dynamics of DNA methylomes underlie oyster development
Source: PLoS Genet. 2017 Jun 8;13(6):e1006807. doi: 10.1371/journal.pgen.1006807 (PMC5481141; doi:10.1371/journal.pgen.1006807)
Supplement: S2 Table — The RNAseq counts in Zhang et al. (RPKM values given in Table S14 of that paper) [42] were averaged as indicated. (DOCX) [file pgen.1006807.s007.docx]

| **Methylation (This study)** | **Expression (Zhang et al.)** |
| --- | --- |
| Oocytes | E (Egg) |
| 2/8 Cells | TC (Two Cell embryos) |
|  | FC (Four Cell embryos) |
| Morula | EM (Early Morula) |
|  | M (Morula) |
| Blastula | B (Blastula) |
|  | RM (Rotary Movement) |
|  | FS (Free Swimming) |
| Gastrula | EG (Early Gastrula) |
|  | G (Gastrula) |
| Trochophore | T (Trochophore) 1 |
|  | T2 |
|  | T3 |
|  | T4 |
|  | T5 |
| D larvae | ED (Early D larvae) 1 |
|  | ED2 |
|  | D (D larvae)1 |
|  | D2 |
|  | D3 |
|  | D4 |
|  | D5 |
|  | D6 |
|  | D7 |
| Spat | S (Spat) |
